# Increased women’s empowerment and regional inequality in Sub-Saharan Africa between 1995 and 2015

**DOI:** 10.1371/journal.pone.0272909

**Published:** 2022-09-14

**Authors:** Erica M. Rettig, Robert J. Hijmans

**Affiliations:** Department of Environmental Science and Policy, University of California, Davis, Davis, California, United States of America; LSU Health Sciences Center New Orleans: Louisiana State University Health Sciences Center, UNITED STATES

## Abstract

Women’s empowerment is a fundamental human right but attempts to measure progress in this area have been limited. We used 142 nationally representative surveys to quantify empowerment in six domains (Intimate Partner Violence, Family Planning, Reproductive Healthcare, Employment, Education, and Decision-Making) for first-level subdivisions of all countries in Sub-Saharan Africa for three years (1995, 2005, and 2015). The possible value for each domain ranged between zero (worst) and one (best). The median value for employment decreased by 0.02, but it increased between 0.09 and 0.16 for the other domains. The average empowerment score increased from 0.44 to 0.53, but it remained low for Education (0.34). While progress was clear and consistent, it was uneven within and between countries, and Sahelian West Africa fell further behind. The expanded understanding of geographic variation and trends in women’s empowerment that we provide should be instrumental in efforts to improve women’s lives.

## Introduction

Gender equality is a fundamental human right and it is a component of several categories of the United Nations Sustainable Development Goals (SDGs) [1, 2]. Women’s empowerment, the expansion of women’s ability to make strategic life choices [3], has been shown to be beneficial to women and entire families [4–6]. For example, it has been linked to improved child nutrition [7–9], higher food security [10], lower child morbidity and mortality rates [11, 12], and the use of clean household fuel [13].

To better understand opportunities for and obstacles to women’s empowerment, we need to know how it changes over time and space, and what drives these changes. However, empowerment is a complex concept that is not easy to measure. Common shortcomings in its measurement are a lack of conceptual rigor; imprecise and biased measurements; and insufficient capture of the multiple dimensions of empowerment [6]. The variation in definition, operationalization, and measurement of empowerment makes it difficult to compare results from different studies [6]. Efforts to quantify empowerment for large areas (e.g. all countries of the world, or a continent) have mostly relied on data that was readily available but not always relevant to the lives of everyday women, such as the proportion of members of parliament that are female [14–16] and insufficiently consider important aspects of women’s empowerment [17]. Moreover, these efforts used mostly national-level data, which can hide important within-country variation [18–21].

Kabeer [3] recognized three dimensions of empowerment: enabling resources; agency; and achievements (outcomes). Two types of agency are recognized: the ability to exercise choice in the household (instrumental agency) and the expression of equitable gender beliefs and attitudes (intrinsic agency) [17]. These three dimensions are inter-related. For example, improved educational attainment is not very useful in the absence of access to high-quality employment for women, which may make families less likely to send their daughters to school [4]. Literacy has been linked to improved child health and access to reproductive healthcare [22], and employment can be related to intimate partner violence (IPV) [23, 24].

We used the resources-agency-outcomes framework to examine subnational variation in aspects of female empowerment across Sub-Saharan Africa (SSA) for three years (1995, 2005, and 2015) using data from 142 Demographic and Health System (DHS) surveys [25], with a total of 2,220,919 individual respondents. We measured women’s empowerment in six domains across the different dimensions of empowerment: Access to Family Planning and Access to Reproductive Healthcare (resources); Intimate Partner Violence (intrinsic agency), Decision-Making (instrumental agency); and Educational and Employment (outcomes). For each domain, scores between 0 (lowest possible empowerment) and 1 (highest possible empowerment) were computed based on the answers to one or more survey questions. Employment and Education scores were adjusted for the inequality of women relative to men to distinguish poor outcomes for men and women, perhaps due to poverty, from situations where men had better outcomes than women.

Our objective was to better understand spatial and temporal variation in important aspects of empowerment across SSA. Because of the contested nature of how female empowerment should be measured [6, 17, 26], and to allow for comparison with future work [6], our focus is on the six individual domains, as these are conceptually relatively straightforward, and not on an overall empowerment score. Nevertheless, we also averaged the domain scores to summarize the trends in the domains of women’s empowerment that we observed. This score is referred to as the Female Empowerment Index (FEMI) [13].

## Results

### Continental patterns

In 2015, the (population-weighted) median scores for the empowerment domains were between 0.34 (Education) and 0.66 (Reproductive Healthcare; Table 1). The score for five out of the six domains increased between 0.10 and 0.15 between 1995 and 2015. Progress was most pronounced in agency related domains: the Decision-Making score increased by 0.15 and the Intimate Partner Violence score increased by 0.11. However, the lower quantile of Decision Making did not change much, so the inequality in this domain also increased considerably. The resources related domains increased with 0.10 or more, but the Family Planning score of 0.51 was much lower than the scores for Reproductive Healthcare and the agency related domains that were all 0.61 or higher. The lowest scores were in the outcome domains. While Education improved with 0.1, all other domains bar Employment had an increase of at least that much, and its 2015 score of 0.34 remained very low. Employment was the only domain with a score that decreased.

**Table 1:** Female empowerment over time. Median and 10th-90th percentile ranges (in parentheses) for six female empowerment domains and the Female Empowerment Index, for all women in SSA for three years (1995, 2005, and 2015).

	1995	2005	2015
*Resources*			
Family Planning	0.38 (0.14–0.71)	0.43 (0.21–0.76)	0.51 (0.19–0.81)
Reproductive Healthcare	0.56 (0.23–0.81)	0.58 (0.28–0.84)	0.66 (0.41–0.87)
*Agency*			
Decision-Making	0.47 (0.23–0.57)	0.49 (0.22–0.74)	0.62 (0.25–0.87)
Intimate Partner Violence	0.50 (0.28–0.72)	0.52 (0.30–0.77)	0.61 (0.42–0.83)
*Outcomes*			
Education	0.24 (0.02–0.77)	0.25 (0.04–0.84)	0.34 (0.07–0.88)
Employment	0.48 (0.32–0.64)	0.49 (0.27–0.68)	0.46 (0.32–0.68)
Female Empowerment Index	0.44 (0.26–0.65)	0.46 (0.31–0.70)	0.53 (0.33–0.77)

The FEMI increased by 20%, from 0.44 in 1995 to 0.53 in 2015. Most empowerment domains were strongly correlated (Table 2), except for employment, which was very weakly correlated with the other domains (0.03 to 0.18).

**Table 2:** Association between female empowerment domains. Pearson’s correlation coefficient for first-level administrative subdivisions for 39 countries in SSA, using the most recent survey data available for each country (n = 531,047).

	Intimate Partner Violence	Family Planning	Reproductive Healthcare	Employment	Education
Family Planning	0.54				
Reproductive Healthcare	0.70	0.62			
Employment	0.15	0.08	0.18		
Education	0.61	0.79	0.60	0.15	
Decision- Making	0.54	0.42	0.36	0.03	0.42

The decline in the Employment domain was due to an increase in inequality between men and women in that domain. Although a higher proportion of women had some form of employment in 2015 than in 1995 (0.56 vs 0.51), after adjusting for inequality, the median score dropped from 0.48 to 0.46. In the other inequality-adjusted domain, Education, there was also an increase in inequality between 1995 and 2015. Completion of primary school went from a ratio of 9.5 women per 10 men in 1995 to 8.4 women per 10 men in 2015. Education was also the only domain where the median value of 0.34 in 2015 (Table 1) was much lower than the mean value of 0.41. For all other domains, the difference between the median and mean scores were +/- 0.03.

### Geographic inequality

**Fig 1:**
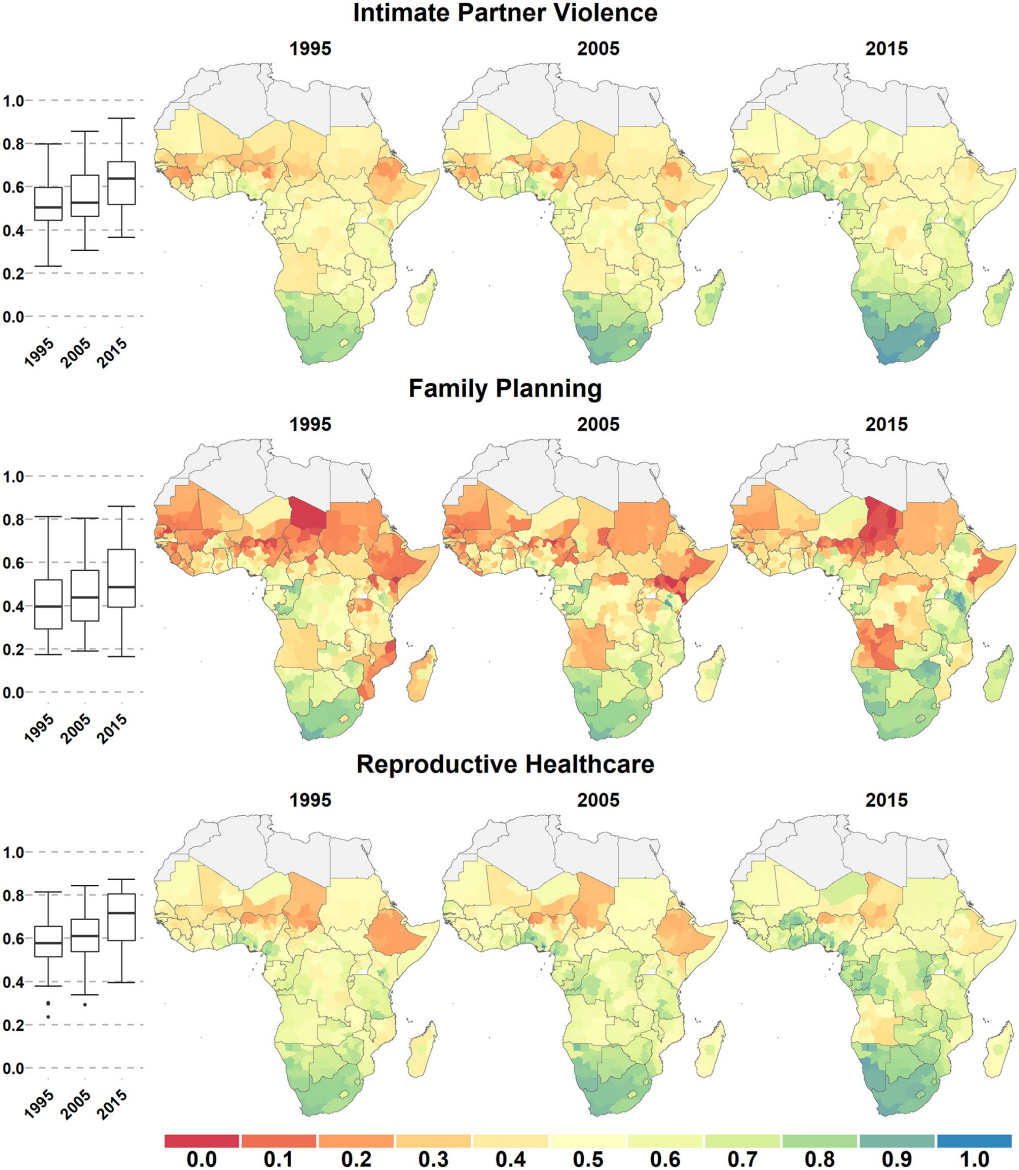
Intimate Partner Violence (a), Access to Family Planning (b), and Access to reproductive healthcare(c). Scores for three female empowerment domains across Sub-Saharan Africa in 1995, 2005, and 2015. Scores can range from 0 (no empowerment) to 1 (full empowerment) and are shown for first-level administrative subdivisions. Boxplots (left) show the range and quartiles scores for each domain.

**Fig 2:**
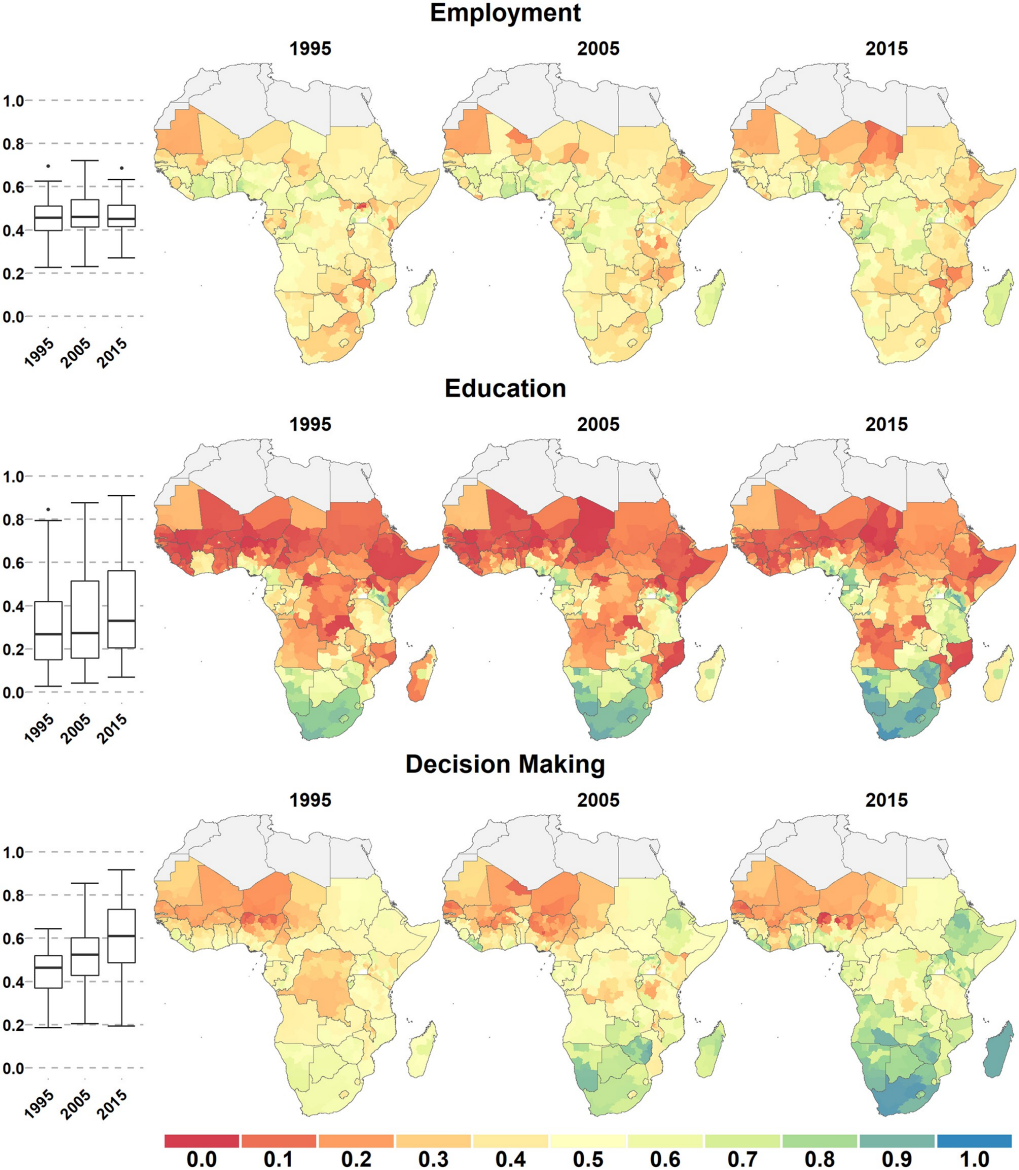
Employment (a), Education (b), and Decision-Making (c). Scores for three female empowerment domains across Sub-Saharan Africa in 1995, 2005, and 2015. Scores can range from 0 (no empowerment) to 1 (full empowerment) and are shown for first-level administrative subdivisions. Boxplots (left) show the range and quartiles scores for each domain.

**Fig 3:**
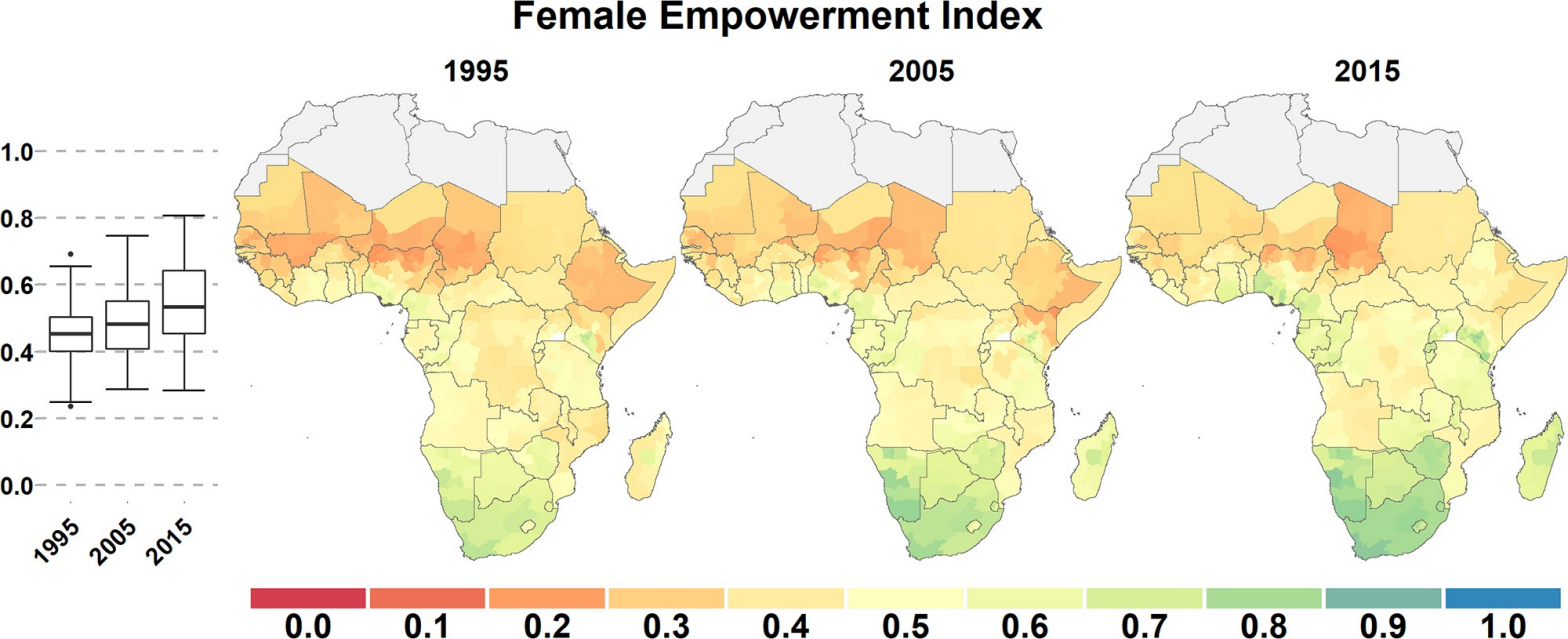
The Female Empowerment Index. Scores for first-level administrative areas in Sub-Saharan Africa in 1995, 2005, and 2015. This is the median value for the six domains shown in Figs 1 and 2. Boxplots (left) show the range and quartiles scores for each domain.

In all empowerment domains except employment, there was a continental geographic gradient from north to south, with scores increasing when going southward. More inland regions also tended to have lower scores than coastal regions, forming a T-shaped band of lower scores across the continent (Figs 1–3). These general patterns were relatively stable across domains and over time.

Geographic inequality was the highest in Education, with both the lowest 10th percentile (0.07) and the highest 90th percentile score (0.88) in 2015. It was also very high for Family Planning (0.19 to 0.81). Geographic inequality was least pronounced in employment, with a 10th percentile score of 0.32 and a 90th percentile score of 0.68 in 2015. Geographic inequality strongly increased between 1995 and 2015 for the Decision-Making domain: the scores did not change much in Sahelian West Africa, while it increased considerably in southern Africa and in Ethiopia. Geographic variation in the FEMI increased between 1995 and 2015 because of the decline or small increase of domain scores in northern and inland areas, while they increased much more in southern and coastal SSA.

For all domains (except Employment) there were marked geographical differences in the magnitude, or even the direction, of change over the period of study (S1 and S2 Figs). For example, increases in the Decision-Making domain averaged 0.34 in southern SSA but only 0.03 in northern SSA. There were remarkable gains in Access to Family Planning in eastern Africa (e.g., Rwanda increased by 0.41, Ethiopia 0.39, and Malawi 0.38), but it decreased or remained stagnant in much of Central African and saw large declines in Sahelian west Africa.

### Subnational variation

**Fig 4:**
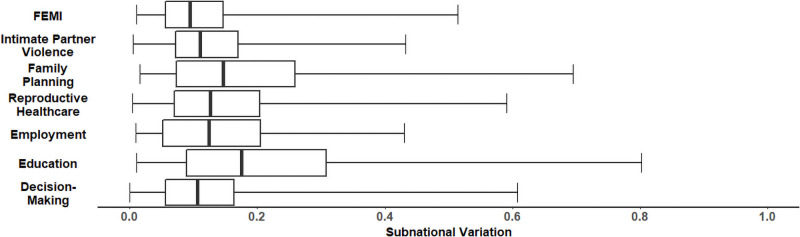
Subnational variation. Subnational variation in the Female Empowerment Index (FEMI) and the six empowerment domains across 39 countries in SSA. Subnational variation for each country was expressed as the difference between the 10th and 90th percentile scores for first-level subdivisions. Boxplots express the range of variation between countries.

We computed the median inter-quantile range for the 10^th^ to 90^th^ percentile scores as of 2015 for each country to quantify the amount of subnational variation within each domain. The median range size (across countries) was between 0.10 (Decision-Making) and 0.18 (Education) for the six domains and was 0.10 for the FEMI (Fig 4). Subnational variation was relatively low in southern Africa and especially high in Nigeria, Kenya, Ethiopia, Cameroon, and Uganda (Figs 1–3). For example, in 2015, the FEMI inter-quantile range was 0.51 for Nigeria and 0.33 for Kenya.

### Association with national-level indicators

**Fig 5:**
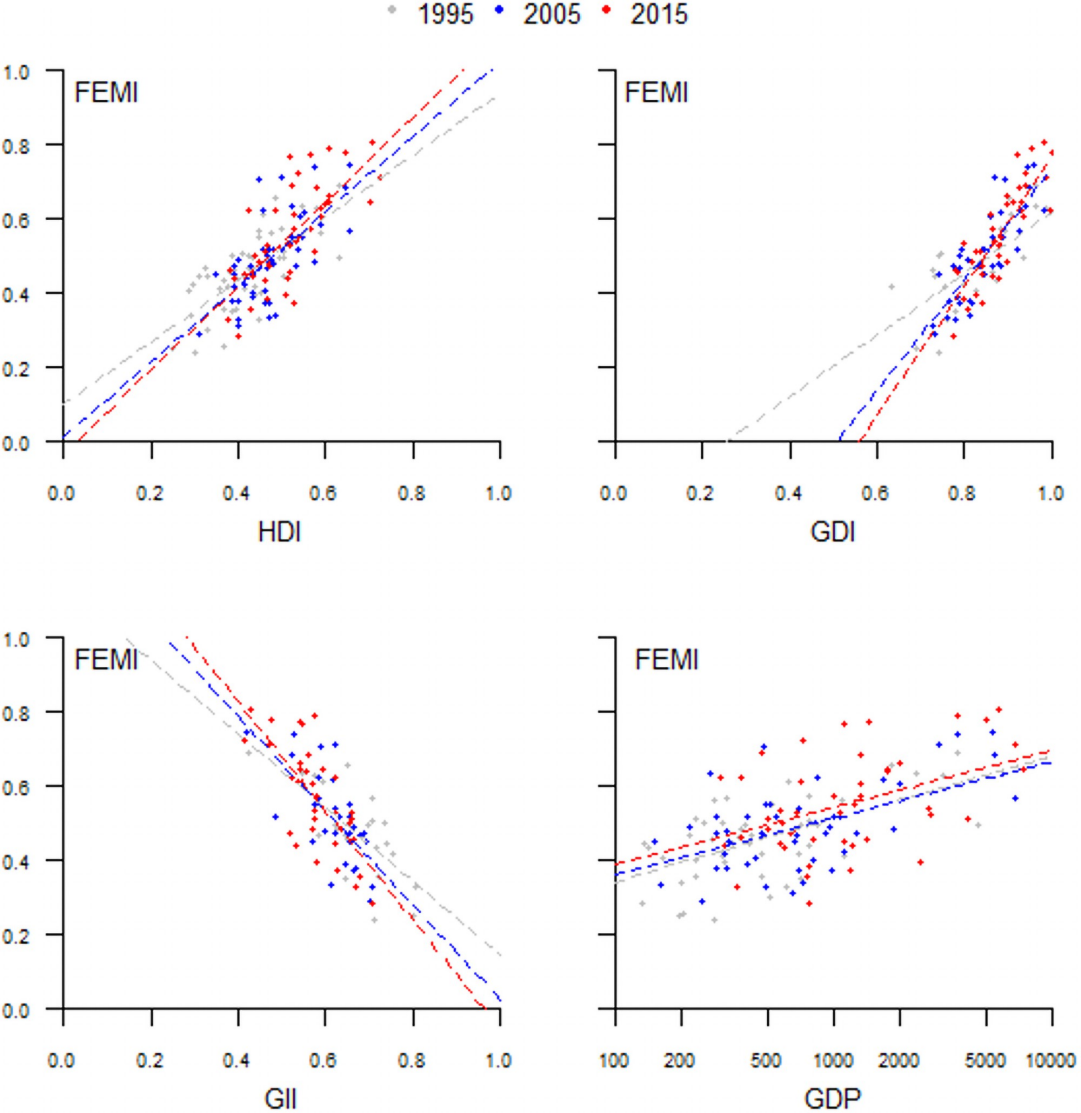
Relationships between development indices. The Human Development Index (HDI), the Gender Development Index (GDI), the Gender Inequality Index (GII), and Gross Domestic Product (GDP, log-scale) versus the Female Empowerment Index (FEMI). National level data for countries in Sub-Saharan Africa in 1995, 2005, and 2015.

The correlation coefficient between the FEMI and four prominent national-level indicators of wellbeing for the three years of the study was 0.70 for the Human Development Index (HDI), 0.79 for the Gender Development Index (GDI), and -0.71 for the Gender Inequality Index (GII) (Fig 5). The effect of log-transformed per capita GDP on the FEMI was positive but small (Fig 5). While the slope of the regression line was essentially the same for the three years, the intercept was slightly higher for 2015. This suggests a very small improvement in FEMI for a given increase in GDP. The correlation between these two variables decreased over time, with scores of 0.52 in 1995, 0.49 in 2005, and 0.41 in 2015. The Sahelian countries generally had the largest negative model residuals for GDP, indicating that these countries have a noticeably lower FEMI than expected given the other national-level indices. In contrast, the southern African countries and Kenya did better than expected (S3 Fig).

## Discussion

We quantified changes in domains of woman’s empowerment for first level national subdivisions across Sub-Saharan Africa. We showed that there was clear and consistent progress in most domains of women’s empowerment, but also that empowerment is very heterogeneous at the continental scale and national scales, and that geographic inequality in empowerment has increased between 1995 and 2015.

We found that the scores for the two empowerment enabling resources related domains strongly improved at the continental scale. However, there were stark differences between and within countries in the Access to Family Planning domain. Its score generally increased in South and East Africa, but it stagnated or declined in many other countries. This occurred despite efforts to improve access during the study period [27, 28]. It is not clear whether such programs are too limited in scope, ineffective, or the degree to which their success is affected by changing attitudes towards contraception. Additional survey data related to the reasons for not using contraception could be used to clarify this [29]. In contrast, access to reproductive healthcare increased across the continent and this domain had the highest score in all three years considered.

Both agency-related domain scores increased, but while the Intimate Partner Violence score consistently increased across the continent, that was not the case for Decision-Making. Decision-Making had the highest overall increase, thanks to strong increases in Southern Africa and elsewhere, and despite the lack of change in Sahelian west Africa.

Educational attainment is a key outcome dimension of women’s empowerment [30–32]. Even though access to education (a resource) can be directly influenced through policy, it stood out for both high inequality between regions and very low attainment overall, in part because of increasing gender inequality. Within-country educational attainment is extremely heterogenous and consideration of national values alone is inadequate because it can lead to inefficient policy and allocation of resources.

Employment did not show clear spatial or temporal patterns and therefore appeared to be the least informative of all domains. Surprisingly, while the other domains were strongly correlated with each other, employment was very weakly correlated with the other domains and the correlation with Education was unexpectedly low. This may be because most reported employment was in the informal sector, where education may be less relevant, but further examination of this finding is warranted.

The explicit consideration of subnational variation should help shed more light on the processes that shape women’s educational attainment and empowerment in general. For example, the northern parts of the Sahelian countries tend to be more similar to each other than to more southern regions in the same countries. This suggests that the processes that shape empowerment in this region are partly independent of national policies and could be better understood by regional analysis, and that the domains are reasonably measurement-invariant across countries (Miedema et al., 2018). Such regional patterns were also found for differences between assets of male and female headed households [33]. They also illustrate that a focus on expanding access to education may be insufficient to solve regional and social educational inequalities. Programs intended to increase education outcomes need to consider subnational inequalities and put additional emphasis on ensuring that girls attend school [34, 35].

The Sustainable Development Goals [36] provide a global focus on several aspects of international development, and research is urgently needed to assess progress towards these goals [37]. Our methods can contribute to a better understanding of patterns of change within several of the SDGs. Target 3.7 (universal access to family planning) directly relates to the Access to Family Planning domain. The Reproductive Healthcare domain connects to target 5.6 (universal access to reproductive health and rights), as well as targets 3.1 (reduce maternal mortality) and 3.2 (reduce infant and under-5 mortality) via the connection between maternal healthcare and maternal mortality ratio and child health [38, 39]. Goal 4’s topic is universal and equitable education, which relates directly to the Education domain. Physical, sexual, and emotional violence (targets 5.1–5.3) are addressed in the IPV and Decision-Making domains. Employment is connected to target 8.5 (universal employment), and our results are also relevant for target 10.2 (socially, politically, and economically empower all people, regardless of personal characteristics).

Empowerment is a complex concept that cannot be perfectly quantified. A single empowerment index will likely be contested, although some progress has been made in rigorously defining standardized measures [17]. For this reason, we focused on a number of domains that are important examples of resources, agency and outcomes; and not on an overall index; even though we used one to summarize our findings. Additional domains might be considered, to the extent that data is available. But the strong correlation between our results and other gender related indices demonstrates that they broadly capture the same patterns at the national level [16]. The differences between these indices merit additional exploration, but their correlation suggests that the conceptual shortcomings of the variables chosen in these national-level indices may be less important than previously argued [14, 15, 37, 40]. Future work could explore these associations by understanding patterns within data-rich countries to enable better estimation for data-poor countries. The weaker correlation of the FEMI with GDP suggests that while increasing wealth helps to improve women’s empowerment in the poorest countries, this effect decreases as countries become wealthier. Importantly, it also suggests that a high GDP is not required to improve empowerment.

The data we used are not perfect, and numbers for individual sub-national areas, and for some countries are uncertain. While future work will undoubtedly refine our results, we have shown is that quantitative approaches can be an important tool in this field of research. The consistency of the spatial and temporal patterns we show, even though they are derived from numerous independent surveys, provides strong support for their validity. Quantitative and spatially disaggregated approaches like ours may help avoid overly generalized statements, and help frame more in-depth studies of empowerment, whether quantitative or qualitative.

## Methods

### Overview

We compiled all available DHS Standard Survey data for countries in sub-Saharan Africa as well as DHS Continuous Survey data for Senegal that were available as of October 2020 (ICF, 1989–2019; S1 Table). The Burundi 1987 survey was not used because data were not available for administrative subdivisions. We were unable to obtain access to the surveys for Eritrea and some of the surveys for Equatorial Guinea and South Africa.

The empowerment domains were created by grouping related questions or groups of questions on different topics (Table 3). All responses were standardized to binary answers with zero representing disempowerment and one representing empowerment, except for the Reproductive Healthcare and Employment domains, which have additional intermediate values between zero and one.

**Table 3:** Survey questions. Survey questions used and their range of possible response values, by domain.

Domain	Question	Possible Values
Decision-Making	Does the respondent have a say in her health?	0.0, 1.0
	Does the respondent have a say in large purchases?	0.0, 1.0
	Does the respondent have a say in visits to family?	0.0, 1.0
	Does the respondent have a say in food to be cooked?	0.0, 1.0
	Does the respondent have a say in deciding what to do with money?	0.0, 1.0
Employment	Did the respondent work all year, part of the year, or not at all?	0.0, 0.5, 1.0
	Was the respondent paid cash, mixed cash and in-kind payments, in-kind payments only, or not at all?	0.0, 0.5, 0.75, 1.0
Education	Did the respondent attend at least six years of school?	0.0, 1.0
	Can the respondent read a short paragraph shown to them?	0.0, 1.0
Intimate Partner Violence	Was the respondent married before the age of 18?	0.0, 1.0
	Is beating justified if the respondent goes out without telling her partner?	0.0, 1.0
	Is beating justified if the respondent neglects the children?	0.0, 1.0
	Is beating justified if the respondent argues with her partner?	0.0, 1.0
	Is beating justified if the respondent refuses to have sex?	0.0, 1.0
	Is beating justified if the respondent burns the food?	0.0, 1.0
Reproductive Healthcare	How many prenatal visits did the respondent have for her children born within the last three years?	0.0, 0.5, 1.0
	Were the respondent’s children born within the last three years delivered by a professional?	0.0, 1.0
	Did the respondent have a post-natal visit within two months of the birth of children born within the last three years	0.0, 1.0
	Did the respondent have a child before the age of 18?	0.0, 1.0
Family Planning	Is the respondent using modern contraceptive methods if they are married and do not desire children within the next two years?	0.0, 1.0

For each survey, responses from individual women were aggregated to the first administrative level below the country level (such as “states” or “provinces”). Some surveys, particularly early ones, used custom regions or regions that do not match current first administrative level boundaries. In these cases, we used interpolation to estimate the values for the current first administrative level areas [18, 19].

For the sexual violence category and the Reproductive Health and Education domains, we assessed the possibility of age-cohort effects due to the large age range of the survey (e.g. a woman aged 49 who was married at age 16 was married 33 years before the survey, and her experience may not reflect the experiences of younger women as attitudes and practices change). We found that the results were very similar results for women aged 18–30 versus women aged 18–49 when comparing the survey data (0.0, -0.01, and +0.03, for sexual violence, Reproductive Health, and Education, respectively), so we opted to not restrict the sample by age in order to preserve a larger sample size.

### Domain scores

#### Intimate Partner Violence (IPV)

The IPV domain has two equally weighted categories: physical violence and sexual violence. For physical violence, we used five questions where women say whether it is acceptable for their partner to physically abuse them in different circumstances (e.g., “Do you believe beating is justified if the respondent argues with her partner?”; Table 3). Sexual violence is indicated by adolescent marriage because it indicates a lack of sexual decision-making power and because childhood marriage is considered sexual violence in and of itself [41].

It should be noted that DHS surveys include questions on women’s direct experience with physical and sexual violence. However, prior research showed that these direct indicators are substantially lower than reported rates from other surveys [18, 42, 43], possibly due to respondents being hesitant to report on potentially traumatic or sensitive topics to a stranger [44]. Because of this, we opted for assessing attitudes towards IPV to represent physical violence rather than its direct incidence.

#### Employment

The employment domain examines both the regularity of work and the type of payment received (Phan, 2016). We consider both formal and informal work, the latter of which is an important source of income for lower-income women [45].

For regularity of work, respondents were assigned a 1 if they reported having year-round employment, 0.5 if they reported part-time or seasonal employment, and 0 if they had no employment. For the pay portion of the domain, respondents were assigned a 1 if they were paid in cash, 0.75 if they were paid a mixture of cash and in-kind payments, 0.5 if they were paid only in-kind, and 0 if they were not paid. The results for these questions were averaged and aggregated for women and men separately.

The Employment (and Education) domains were adjusted for inequalities between men and women by multiplying the woman’s score by the inequality ratio (the woman’s score divided by the men’s score). This lowers the adjusted score if men have higher scores than women but not if both men and women have low scores. In this way, the scores of poor areas where few people of either gender may be employed or educated are not affected, but the scores are affected for areas with true inequality, where men are employed or educated at higher rates than women.

#### Education

The education domain is the average of two scores: the proportion of women who have completed at least six years of schooling, and the proportion who can read a simple paragraph in their native language without difficulty. The education domain is adjusted identically to the employment domain to help distinguish between regions where there is little schooling from true gender inequality.

#### Reproductive Healthcare

The Reproductive Healthcare domain assesses whether mothers and their children receive adequate healthcare services and whether women had children as adolescents, which is associated with higher mortality and morbidity rates [41]. The World Health Organization recommends that all pregnant women receive four antenatal visits carried out by a trained worker, that children are delivered in a professional setting, and that infants have at least one postnatal visit within two months after birth [46].

For the antenatal care, child delivery, and postnatal care questions, we counted all children born within three years of the date of the interview. The adolescent childbearing, delivery, and postnatal visit categories were calculated in the standard fashion (that is, with 0 representing disempowerment and 1 representing empowerment). Antenatal visits were calculated using intermediate values to represent women who had some care, but less than the recommended amount of care: women who had no professional antenatal visits were assigned a 0, women with between one and three visits a 0.5, and women with 4 or more visits a 1. The overall Reproductive Healthcare domain is the average of these four categories.

#### Decision-Making

For Decision-Making, we computed the average value of the answers to four questions (Table 3). The domain score is the mean of the answers to these questions. For this domain, “self” and “self and partner” were both assigned a 1, representing empowerment, as the woman had a say in the decision-making for that question.

We included two less critical choices within the decision-making domain: whether women have a say in what to cook for dinner, and whether they have a say in household purchases (“large purchases” are addressed in a separate question). We included these because even though the ability to make such choices may not greatly affect a woman’s life, a lack of ability to make decisions over even basic choices clearly indicates disempowerment.

#### Access to family planning

The Access to Family Planning domain considers whether married women who do not wish to currently become pregnant are using modern contraceptives. DHS survey data include a pre-calculated version of access to family planning, which measures the proportion of married women who are currently using modern contraceptive methods. However, in many cases, calculations using the programming code released by DHS [47] that follows the methods of Bradley and Croft [48] do not match the already-calculated version [18]. We corrected the calculations, and we also changed the denominator from "all married women" to "married women who do not want a child at the time of the survey". Excluding married women who currently want children, and therefore do not need contraception, more accurately captures effective access for empowerment purposes.

#### Estimation procedures

To create yearly estimates at the first administrative level for every country in sub-Saharan Africa, we followed a three-step process. First, we estimated missing data at the first administrative level (“imputation”). Second, for surveys that did not use the first administrative level for their surveys, we downscaled the results to the first administrative level (“interpolation”). In a few cases, data were given at the second administrative level. In these cases, we aggregated them to the first administrative level to ensure a large enough sample size for each area. Finally, we used the corrected, standardized survey data to create estimates for 1995 through 2015 (“extrapolation”), although in this paper we focus on general trends by examining in detail the years 1995, 2005, and 2015.

### Imputation

Although the DHS surveys are highly standardized, a given survey does not necessarily include all questions, particularly for earlier surveys. If questions were missing, we estimated them using Random Forest [49], using standardized predictor variables that were available for all surveys (year, country, age at first marriage, age at first child, number of years of education, longitude and latitude, age at the time of the survey, and number of births in the last five years). For education questions, “number of years of education” was dropped as a predictor.

Two domains (education and employment) used adjustments in order to capture gender-based inequalities in these domains. In these cases, the men’s domains were estimated separately from the women’s, using the predictor variables of year, longitude, and latitude (other potential predictor variables for men were missing from one or more surveys).

The number of areas needing imputation varied by domain, ranging from less than 1% for Access to Family Planning to 26% for men’s employment (Table 3). The Random Forest model R^2^ values ranged from 0.56 in the case of men’s employment to 0.84 for IPV and Education (Table 4). Once estimations for all domains were imputed, FEMI scores were calculated for each area as the arithmetic mean of the six domain scores.

**Table 4:** Estimation model quality. R^2^ values for the Random Forest model used for imputation, the proportion of first-level administrative subdivisions for which values were imputed, and R^2^ values for the Random Forest model used for extrapolation, by first administrative area. N/As for men’s employment and education extrapolation is because inequality adjustments were done before extrapolation. The FEMI was calculated after imputation so that all domains were available for each region, so no fraction is imputed for FEMI during extrapolation.

Domain	R^2^ (imputation)	Proportion Imputed	R^2^ (Extrapolation)
Decision-Making	0.76	0.27	0.85
Employment (Women)	0.56	0.13	0.61
Employment (Men)	0.60	0.16	n/a
Education (Women)	0.87	0.25	0.89
Education (Men)	0.72	0.26	n/a
Intimate Partner Violence	0.84	0.26	0.83
Reproductive Healthcare	0.81	0.03	0.86
Family Planning	0.71	0	0.78
Female Empowerment Index	n/a*	n/a*	0.89

### Interpolation

DHS surveys generally include either geographic coordinates or information on the first administrative level where the survey was located. However, some surveys, particularly older ones, used different geographic boundaries (typically aggregations of multiple first administrative areas). In addition, in several countries, the subdivision boundaries changed over time. To standardize results, all values were calculated for the current first administrative areas for each country.

Interpolation was achieved in one of two ways. In the case where there was a survey before and after a non-standard survey, we used the year-weighted mean of the surveys immediately before and after the non-standard survey and applied a linear adjustment factor to ensure that the overall regional mean matched that of the original survey regions.

In the case where only there was only one predictor survey available, we first aggregated the first administrative level survey values based on what region it belonged to in the regional survey. Then, for each domain, we calculated the difference between the aggregated regions of the regional survey and the created regions of the first administrative level survey. These differences were then used to downscale the larger regions to the first administrative level areas. Additionally, there was one special case. In Mali, there were two surveys: one in 1987, and one in 2012, but the earlier survey was only done in the southern half of the country. In this case, we split the north and south and treated them as different countries for interpolation purposes.

### Extrapolation

To estimate the yearly values for each first administrative level for each country, we used one of three methods, depending on the available survey data. If a survey was within 2 years of 1995, 2005, or 2015, we directly used the survey by reassigning it to the relevant year. In other cases, if there were two or more surveys, we used the linear trend to estimate values for each domain and year of interest. If there were no surveys or only one survey was available, we created a Random Forest model to predict values for each of the three years. We used UN-reported Human Development Index and maternal mortality values as country-level predictors, and UN-adjusted population density and survey data where available as first administrative level predictors.

### Further analyses

To assess the amount of subnational variation between countries, we calculated the 10^th^ to 90^th^ percentile range of scores as well as median values within each country for each domain and FEMI. We then compared these values for each country. To assess the degree of association between FEMI and national level development indicators, we used linear regression models of FEMI in response to the Human Development Index, the Gender Development Index, the Gender Inequality Index, and log-scaled Gross Domestic Product per capita.

## Supporting information

S1 Fig Change in Female Empowerment Index and domain scores between 1995 and 2015. For first-level administrative subdivisions in Sub-Saharan African countries.
(PDF)

S2 Fig The number of domains experiencing a decline in value between 1995 and 2015. For first-level administrative subdivisions in Sub-Saharan African countries.
(PDF)

S3 Fig Model residuals for linear regression models. Models of the Female Empowerment Index as a function of the Human Development Index (HDI), Gender Development Index (GDI) Gender Inequality Index (GDI), and the Gross Domestic Product GDP), for each country in sub-Saharan Africa in 2015. Countries colored in white indicate that the corresponding variable was not available for that country.
(PDF)

S1 Table Survey data used, by country and year. All survey data are Demographic and Health Surveys (https://dhsprogram.com/).
(PDF)
