# Supplementary material for: Increased women’s empowerment and regional inequality in Sub-Saharan Africa between 1995 and 2015
Source: PLoS One. 2022 Sep 14;17(9):e0272909. doi: 10.1371/journal.pone.0272909 (PMC9473440; doi:10.1371/journal.pone.0272909)
Supplement: S2 Fig — For first-level administrative subdivisions in Sub-Saharan African countries. (PDF) [file pone.0272909.s002.pdf]

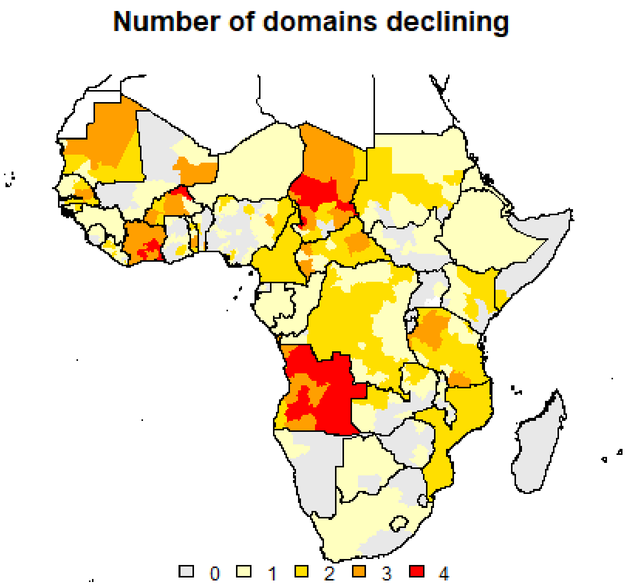

S2 Fig. The number of domains experiencing a decline in value between 1995 and 2015. For first-level administrative subdivisions in Sub-Saharan African countries.
