# Supplementary material for: Increased women’s empowerment and regional inequality in Sub-Saharan Africa between 1995 and 2015
Source: PLoS One. 2022 Sep 14;17(9):e0272909. doi: 10.1371/journal.pone.0272909 (PMC9473440; doi:10.1371/journal.pone.0272909)
Supplement: S3 Fig — Models of the Female Empowerment Index as a function of the Human Development Index (HDI), Gender Development Index (GDI) Gender Inequality Index (GDI), and the Gross Domestic Product GDP), for each country in sub-Saharan Africa in 2015. Countries colored in white indicate that the corresponding variable was not available for that country. (PDF) [file pone.0272909.s003.pdf]

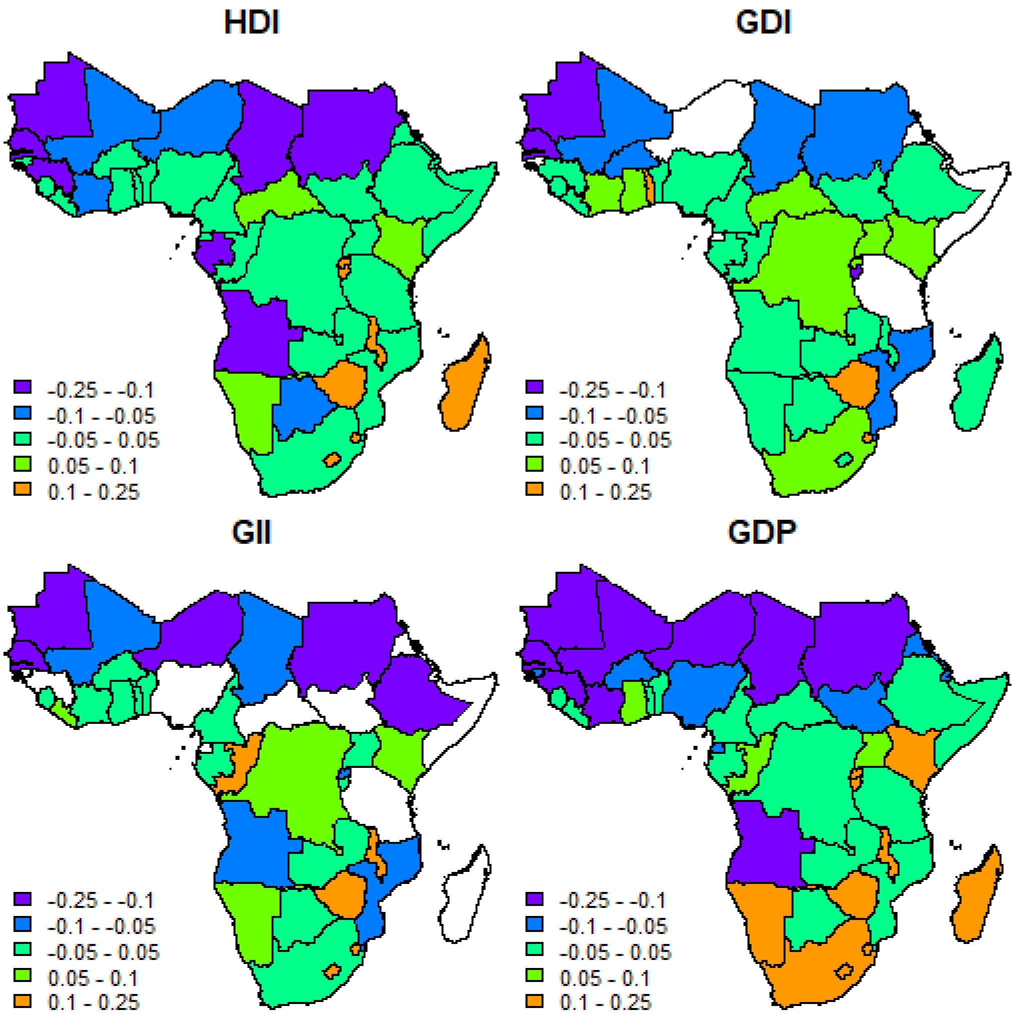

S3 Fig. Model residuals for linear regression models. Models of the Female Empowerment Index as a function of the Human Development Index (HDI), Gender Development Index (GDI) Gender Inequality Index (GDI), and the Gross Domestic Product GDP), for each country in sub-Saharan Africa in 2015. Countries colored in white indicate that the corresponding variable was not available for that country.
