# Supplementary material for: Increased women’s empowerment and regional inequality in Sub-Saharan Africa between 1995 and 2015
Source: PLoS One. 2022 Sep 14;17(9):e0272909. doi: 10.1371/journal.pone.0272909 (PMC9473440; doi:10.1371/journal.pone.0272909)
Supplement: S1 Table — All survey data are Demographic and Health Surveys (https://dhsprogram.com/). (PDF) [file pone.0272909.s004.pdf]

S1 Table. Survey data used, by country and year. All survey data are Demographic and Health Surveys (https://dhsprogram.com/)

Country	Survey Years
Angola	2015
Benin	1996, 2001, 2006, 2011, 2017
Botswana	-
Burkina Faso	1993, 1998, 2003, 2010
Burundi	2010, 2016
Cameroon	1991, 1998, 2004, 2011, 2018
Central African Republic	1994
Comoros	1996, 2012
Côte d’Ivoire	1994, 1998, 2011
Democratic Republic of the Congo	2007, 2013
Djibouti	-
Ethiopia	2000, 2005, 2011, 2016
Equatorial Guinea	-
Gabon	2000, 2012
Gambia	2013
Ghana	1988, 1993, 1998, 2003, 2008, 2014
Guinea	1999, 2005, 2012, 2018
Guinea-Bissau	-
Kenya	1989, 1993, 1998, 2003, 2008, 2014
Lesotho	2004, 2009, 2014
Liberia	1986, 2007, 2013
Madagascar	1992, 1997, 2003, 2008
Malawi	1992, 2000, 2004, 2010, 2015
Mali	1987, 1995, 2001, 2006, 2012, 2018
Mauritania	2000
Mozambique	1997, 2003, 2011
Namibia	1992, 2000, 2006, 2013
Niger	1992, 1998, 2006, 2012
Nigeria	1990, 1999, 2003, 2008, 2013, 2018
Republic Of Congo	2005, 2011
Rwanda	1992, 2000, 2005, 2010, 2014
São Tomé And Príncipe	2008
Senegal	1986, 1992, 1997, 2005, 2010, 2012, 2015, 2016-2019
Sierra Leone	2008, 2013, 2019
Somalia	-
South Africa	1998, 2016
South Sudan	-
Sudan	1989

Swaziland	2006
Tanzania	1991, 1996, 1999, 2004, 2010
Togo	1988, 1998, 2013
Uganda	1988, 1995, 2000, 2006, 2011, 2016
Zambia	1992, 1996, 2001, 2007, 2013
Zimbabwe	1988, 1994, 1999, 2005, 2010, 2015
